# Patient-physician agreement on function and pain is associated with long-term outcomes in sarcoma: findings from a longitudinal study

**DOI:** 10.1007/s11764-023-01473-3

**Published:** 2023-10-17

**Authors:** Urška Košir, Deborah van de Wal, Olga Husson, Nadine Zablith, Robert E. Turcotte

**Affiliations:** 1 Division of Orthopedic Surgery, McGill University Health Centre (MUHC), Montreal, QC Canada; 2 Department of Experimental Psychology, University of Oxford, Oxford, UK; 3 Medical Oncology Department, Netherlands Cancer Institute, Amsterdam, The Netherlands

**Keywords:** Agreement, Sarcoma, Fatigue, Pain, Daily functioning, Quality of life

## Abstract

**Purpose:**

We aimed to describe the level of agreement between patients and physicians on the ratings of daily functioning and pain in a cohort of sarcoma patients and assess how (dis)agreement and its change over time predicted patient-reported outcomes in survivorship.

**Method:**

We performed secondary analysis of longitudinal data from a sarcoma-specialty clinic in Montreal, Canada. Demographics, clinical characteristics and patient-physician agreement were summarized descriptively. Linear mixed models were used to assess the effects of time, baseline agreement, change in agreement over time, interaction of time and change in agreement and 12-month daily functioning, quality of life, and fatigue.

**Results:**

Data were available for 806 patients (57.7% male, x̄ = 53.3 years) who completed at least one questionnaire. Patient-physician disagreement was common on the level of function (43.4%) and pain (45.7%). Baseline physician-patient agreement was associated with better 12-month outcomes. Improvement in agreement on function over time was significantly associated with daily functioning (*F*(2, 212) = 3.18, *p* = 0.043) and quality of life (*F*(2, 212) = 3.17, *p* < 0.044). The pattern was similar though less pronounced for the agreement on pain.

**Conclusions:**

Our study offers novel insights into the importance of patient-physician agreement and communication’s role in long-term patient-reported outcomes in sarcoma.

**Implications for Cancer Survivors:**

The results emphasize the importance of mutual understanding of symptoms and patients’ needs and suggest that further consultation in cases of discordance of ratings and opinions might be beneficial for optimal survivorship.

## Introduction

Health care systems are becoming increasingly patient-centred in which effective communication between the patient and their healthcare providers is key [1]. Communication serves to establish a treatment alliance, facilitates the exchange of information, and promotes patient engagement by shared decision-making [2, 3]. Research to date shows that good and effective communication between a patient and their healthcare providers cannot only lessen the psychological burden of disease, [4] but can also assist comprehension of medical information, promote adherence to treatment, improve satisfaction with care, and subsequently lead to better outcomes [5, 6]. However, effective communication between a healthcare provider and their patient, especially in the field of oncology, is not always achieved [6, 7].

Previous research suggests a lack of concordance or agreement between patients and healthcare providers. For example in symptom reporting, especially subjective symptoms such as fatigue, nausea, and pain, [8, 9] patients tend to report symptoms earlier and more frequently with worse symptom severity compared to their healthcare providers [10, 11]. Similar disagreements have been reported on performance status, survival prognosis and treatment understanding [12–16]. Such disagreements carry important implications and have been associated with worse outcomes, including higher risk of death [16].

Studies that looked at patient-level factors found that disagreements were substantially more common among non-white patients, [13, 17] those with lower educational levels, older age (> 60 years), [14] and low health literacy [18]. While no sex differences were observed between patients’ ratings of their own performance status, oncologists rated women more pessimistically than men, [12] and assigned a better performance status to younger patients and when the disease was aggressive [15]. Physician-level factors can also be associated with better agreement such as age of the physician, physician discomfort or level of training [14, 19].

Patients who might particularly benefit from effective communication with their healthcare providers are those afflicted by rare cancers such as sarcomas. Sarcomas are rare and highly heterogeneous mesenchymal tumours, presenting in virtually any part of the body [20]. Although they account for less than 1% of all adult cancer cases, [21] the impact of this disease on an individual, family, and healthcare system level remains substantial [22]. While the overall survival of patients with cancer is steadily increasing, patients with sarcomas face lower survival rates, with 5-year overall survival ranging 50–65% [22, 23]. Furthermore, they are affected with significantly more medical comorbidities (e.g., cardiovascular diseases, depression, anxiety, asthma) compared to peers without cancer, [24] and up to 50% of the sarcoma patients report a long-term disability status [25]. Patients with sarcomas also report lower quality of life, and suffer from psychological distress more often than patients with other types of cancer [26–29].

In addition to the low rates of research and investment, as well as rare disease status, lack of effective communication may also play a substantial role in the long-term outcomes of patients with sarcoma. Understanding the physician-patient agreement on outcomes such as functioning and pain and their impact on long-term patient-reported outcomes in sarcoma carries important public health and clinical implications for not merely improving these outcomes, but also raising cancer literacy levels in this vulnerable population of patients. By studying a large sample of patients from a sarcoma-specialty center, our study aimed to (1) assess and describe the level of agreement between patients and physicians on the ratings of daily functioning and pain, and (2) evaluate whether the level of (dis)agreement at baseline and change in agreement over time predicted self-reported quality of life, fatigue, and functioning at 12 months of follow-up after the initial surgical treatment.

## Method

### Sample

Interdisciplinary Health Research Team is a database established for collecting information on patients with musculoskeletal neoplasia, including sarcomas. The main purpose of this biobank was to investigate the pathogenesis, treatment, treatment-related morbidity, as well as quality of life and psychosocial outcomes of primary malignant tumors (sarcomas) of the bones and soft tissues. This is a collaborative effort between two major medical centers in Canada, McGill University Health Centre (MUHC), Montreal, and Mount Sinai Hospital, Toronto. The data collection started in 2001 and is still ongoing.

Data for this secondary analysis was retrieved from MUHC site (MUHC Research Ethics Board, reference: 2021–7013). Data is collected prospectively, and questionnaires are administered by the research team during routine appointments at baseline (pre-operative), and subsequently at 3, 6, 12, 24, and 36 months post-operatively. The sample which we requested contained 1,069 individual entries who provided informed consent prior to June 2020. Our analyses are based on 806 patients who have completed at least 1 questionnaire at baseline. We relied on baseline and 12-month assessments because not all outcome measures were collected at 3 or 6 months, and to minimize attrition.

### Measures

#### Demographic and clinical characteristics

Self-reported demographic (e.g. gender, education level) and medical information (e.g. tumor size, grade, and other chronic conditions) was collected at baseline.

### Outcome measures

#### Fatigue

Fatigue was assessed using Functional Assessment of Chronic Illness Therapy (FACIT) – fatigue scale, which is a 13-item scale that asks about fatigue and its impact on social activities, as well as functioning (i.e. sleeping). The scale was originally developed to assess cancer-related fatigue and has shown good reliability and validity in a sample of cancer patients [30]. Cronbach α for of our sample at baseline was 0.82.

#### Functional outcomes

Toronto Extremity Salvage Score (TESS) is a self-reported 31-item questionnaire, developed to measure physical and functional impairment in daily living activities of patients with sarcoma. It includes restrictions in mobility, personal care, and social activities. The sum score is converted to the percentage score which describes physical functioning according to the International Classification of Impairments, Disabilities, and Handicaps (ICIDH) [31]. The measure has been developed and validated for use in patients with sarcoma from age 12 to 80 years [32, 33]. The scale has 2 versions, one for the lower extremity, and one for the upper extremity. For both Cronbach α at baseline was 0.98.

#### Quality of life

Health-related quality of life was assessed with European Quality of Life Questionnaire (EQ-5D), a self-reported questionnaire with two analysis variables. The first is a visual analog scale (VAS), from 0 (worst imaginable) to 100 (best imaginable), which asks patients’ status on the day. The second variables define health across five domains: mobility, self-care, usual activities, pain or discomfort, and anxiety or depression. Each domain is scored on a 3-point Likert scale: no problem (1), some problem (2), and moderate or severe problem (3) [34].

### Musculoskeletal Tumour Society score

Musculoskeletal Tumour Society Score (MSTS) is a physician-reported evaluation of the functional condition and assesses several domains, including pain and functioning on a 0 to 5 scale (0 being the worst, and 5 being excellent) [35].

### Physician-patient agreement

Self-reported and physician-reported functioning and pain scores were extracted from EQ-5D and MSTS scales, respectively. Upon consultation with the physician and PI of the IHRT Biobank, the scores were harmonized as follows: MSTS of 5 corresponded to 1 on EQ-5D, 2–3 to 2, and 0–2 to 3. The Supplementary material shows this in detail.

To describe the physician-patient agreement, two variables were computed: (1) The overall disagreement was a dummy variable (0/1) marking agreement (score 0) when the scores from the EQ-5D and MSTS were the same for the physician and patient, and disagreement (score 1) when the scores were not the same ; (2) The level of agreement was calculated as a difference between the physician and patient score resulting in values ranging from − 2 to 2. Values below 0 described cases where the physician rated one’s function or pain worse than the patient, 0 described no discrepancy thus agreement, and positive values meant that physicians rated one’s function better than the patient themselves.

### Change in agreement over time

The variable to describe change in agreement over time was created by comparing the agreement at baseline with agreement at 12-months. In case of agreement at baseline, but not at 12-months, the cases were assigned “Worsened”, in case of the opposite was true “Improved” and “No change” where no change in agreement was recorded.

### Analysis

Descriptive analyses were used to summarize the sample and levels of agreement. Univariate linear models were performed to assess the associations between the level of agreement and quality of life (VAS), fatigue (FACIT), and daily functioning (TESS). Univariate multinomial logistic regressions (*No change* as reference) were used for associations between demographic variables and change in agreement over time.

Associations between baseline agreement and 12-month outcomes were assessed with linear mixed models. Scores at 12-months (TESS, VAS, FACIT) were entered as dependent variables and time, baseline agreement, change in agreement over time, and interaction of time and change in agreement as fixed effects. Random effect was entered for individual intercept. Models were estimated using Restricted Estimation Maximum Likelihood (REML). Sensitivity analyses revealed no significant pattern in the missing data. Subject’s age, gender, education, and tumor grade were entered as covariates. Patients who were present at baseline but have deceased since enrolling into the study were excluded from the analyses (*N* = 83). Bonferroni correction was used in the post-hoc analyses. Adjusted models are presented in this article, the covariate-unadjusted models can be found in the Supplementary materials.

Analysis were performed using R Studio and GAMlj package [36, 37]. The associated code, sensitivity analyses and all supplementary materials can be accessed at: https://osf.io/bhrwa/.

## Results

### Sample

At baseline, 806 patients (57.7% male) completed at least one questionnaire. Table 1 shows the demographic information and scores on the outcome measures at baseline and 12-months for these patients.

**Table 1:** Demographic and medical information

Overall (*N* = 806)	
**Gender = MALE (%)**	465 (57.7)
**Age (y) (mean (SD))**	53.29 (18.99)
**Tumor type (%)**	
Soft tissue Bone Soft and bone	562 (70.1) 237 (29.6) 3 (0.4)
**Tumor location = Upper (%)**	179 (27.4)
**Tumor grade (%)**	
0 1 2 3 4	70 (10.9) 138 (21.5) 193 (30.1) 234 (36.5) 6 (0.9)
**Marital status (%)**	
Married/Cohabiting Separated/Divorced Single Widowed	445 (63.8) 57 (8.2) 153 (21.9) 43 (6.2)
**Education level (%)**	
Grad school No education Other Primary Secondary University	75 (10.6) 9 (1.3) 6 (0.8) 23 (3.3) 431 (61.0) 162 (22.9)
**Ethnicity (%)**	
Asian Black/Caribbean/African Caucasian/White Hispanic Native Other/Multiple	36 (5.6) 21 (3.3) 515 (80.5) 4 (0.6) 5 (0.8) 59 (9.2)
**BL deceased = Yes (%)**	83 (11.9)
**BL TESS (*N*** **= 653, mean (SD))**	73.51 (20.64)
**12 M TESS (*N*** **= 471, mean (SD))**	79.27 (15.05)
**BL FACIT (*N*** **= 643, mean (SD))**	39.21 (10.71)
**12 M FACIT (*N*** **= 462, mean (SD))**	41.39 (10.89)
**BL VAS (*N*** **= 653, mean (SD))**	72.61 (19.06)
**12 M VAS (*N*** **= 475, mean (SD))**	80.41 (16.25)

*NOTE*: BL = Baseline; 12 M = 12-Months; TESS = Toronto Extremity Salvage Score; FACIT = Functional Assessment of Chronic Illness Therapy; VAS = Visual Analog Scale

### Agreement on function

#### Baseline agreement

Out of 648 patients who had both the self-reported and physician-reported scores on function at baseline, 281 (43.4%) disagreed with their physician at baseline. Of those that disagreed, in 57.3% (161/281) of the cases physician rated patients’ function as worse than patients themselves, and in 42.7% (120/281) of the cases physicians rated patients’ function as better than patients themselves.

### Agreement and 12-month outcomes

**Fig. 1:**
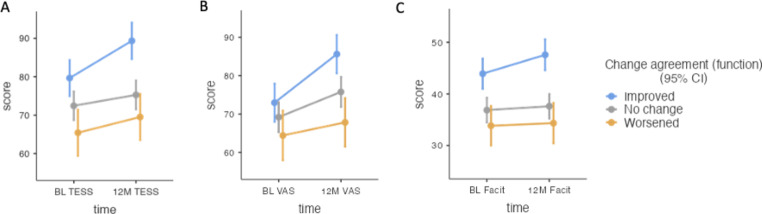
Agreement on function: Time x change in agreement *BL = Baseline; 12 M = 12-months; TESS = Toronto Extremity Salvage Score; FACIT = Functional Assessment of Chronic Illness Therapy; VAS = Visual Analog Scale*.

Three adjusted mixed models were run to assess the baseline agreement on function, change in agreement over time, and their interaction with the main outcomes of interest (i.e., daily functioning, quality of life, and fatigue) at 12-months. Model 1 showed a main effect of time (*F*(1, 212) = 19.25, *p* < 0.001), baseline agreement (*F*(2, 216) = 10.89, *p* < 0.001), change in agreement over time (*F*(2, 211) = 9.28, *p* < 0.001, and a significant interaction of time and change (*F*(2, 212) = 3.18, *p* = 0.043). Daily function significantly improved over time (β = 5.54, SE = 1.26, *p* < 0.001). Patients that agreed with their physician at baseline reported significantly better daily function than those that disagreed, both when the physician rated them better (β = -12.47, SE = 3.20, *p* < 0.001), or worse (β = -13.89, SE = 3.24, *p* < 0.001). For patients whose agreement with the physician improved, the daily function at 12-months improved significantly more than those who had no observed change in agreement (β = 10.64, SE = 3.18, *p* = 0.003), and those who had no change reported significantly better daily functioning compared to those whose agreement with the physician worsened (β = 6.87, SE = 2.73, *p* = 0.02, see Fig. 1).

Model 2 revealed a main effect of time (*F*(1, 212) = 28.89, *p* < 0.001), baseline agreement (*F*(2, 208) = 20.98, *p* < 0.001), change in agreement over time (*F*(2, 210) = 5.41, *p* = 0.005), and a significant interaction of time and change (*F*(2, 212) = 3.17, *p* < 0.044). Overall, quality of life significantly improved over time (β = 7.55, SE = 1.40, p < 0.001). Patients that agreed with their physician at baseline reported significantly better quality of life than those that who were rated as better by their physician (β = -9.19, SE = 3.32, p = 0.018), and those rated as worse (β = -21.47, SE = 3.34, p < 0.001). For those whose agreement with the physician worsened over time, the quality of life at 12-months was significantly lower than those who had no change in agreement (β = 6.35, SE = 2.58, p < 0.001), and those whose agreement improved (β = 13.14, SE = 3.18, p = 0.005, see Fig. 1).

Model 3 revealed a main effect of time (*F*(1, 212) = 4.86, *p* = 0.029), baseline agreement (*F*(2, 211) = 18.12, *p* < 0.001), and change in agreement over time (*F*(2, 218) = 10.61, *p* < 0.001). Overall, fatigue improved over time (β = 1.64, SE = 0.74, p = 0.029). Similarly to quality of life, patients that agreed with their physician at baseline reported significantly less fatigue than those that were rated as better by their physician (β = -6.50, SE = 2.07, p = 0.006), as well as those rated as worse (β = -12.53, SE = 2.08, p < 0.001). Those who improved in agreement over time reported significantly less fatigue than those who had no observed change (β = 8.51, SE = 2.05, p < 0.001), or worsened (β = 11.66, SE = 2.58, p < 0.001). Complete model parameters are presented in Table 2 and the Supplementary materials. Figure 1 shows the change in agreement over time and the scores of daily functioning, quality of life, and fatigue.

**Table 2:** Mixed models for agreement on function

Fixed effects	Model 1: Daily function (TESS)			Model 2: Quality of life (VAS)			Model 3: Fatigue (FACIT)		
	Estimate (SE)	*t*	*p*	Estimate (SE)	*t*	*p*	Estimate (SE)	*t*	*p*
Intercept	75.28 (1.25)	60.23	**< 0.001**	72.63 (1.30)	55.74	**< 0.001**	39.01 (0.81)	48.13	**< 0.001**
Time (BL)	5.54 (1.26)	-4.39	< 0.001*	7.55 (1.40)	-5.37	< 0.001*	1.64 (0.74)	2.20	0.029*
Gender^a^	-1.49 (1.96)	-4.81, 1.82	0.379	-2.94 (1.77)	-1.66	0.098	0.19 (1.10)	0.17	0.864
Age	0.04 (0.05)	-0.05, 0.13	0.392	0.08 (0.05)	1.68	0.094	-0.01 (0.03)	-0.21	0.836
Education	0.96 (1.03)	-1.05, 2.98	0.35	-0.23 (1.08)	-0.21	0.833	0.67 (0.67)	1.01	0.315
Tumor grade	0.31 (0.89)	-1.44, 2.05	0.729	-0.84 (0.93)	-0.90	0.369	-0.88 (0.58)	-1.15	0.130
**Baseline agreement**									
MD lower (ref) – MD higher	1.42 (3.06)	0.47	1.00*	12.28 (3.17)	3.87	< 0.001*	6.03 (1.96)	3.07	0.007*
Agree (ref) – MD higher	-12.47 (3.20)	-3.89	< 0.001*	-9.19 (3.32)	-2.77	0.018*	-6.50 (2.07)	-3.13	0.006*
Agree (ref) – MD lower	-13.89 (3.24)	-4.29	< 0.001*	-21.47 (3.34)	-6.42	< 0.001*	-12.53 (2.08)	-6.01	< 0.001*
**Change in agreement**									
No change (ref) – Improved	10.64 (3.18)	3.35	0 0.003*	6.79 (3.30)	2.06	0.122*	8.51 (2.05)	4.14	< 0.001*
Worsened (ref) - Improved	17.03 (3.96)	4.30	< 0.001*	13.14 (4.13)	3.18	0.005*	11.66 (2.58)	4.53	< 0.001*
Worsened (ref) – No change	6.39 (2.33)	2.74	0.020*	6.35 (2.46)	2.58	< 0.001*	3.15 (1.53)	2.06	0.123*
**Random effects**				**Random effects**			**Random effects**		
ICC	0.391			ICC	0.345		ICC	0.5	
Residual variance	131.1			Residual variance	158.8		Residual variance	41.6	

*NOTE*: *ref* = reference category, meaning that the estimate refers to that in comparison to the other; *MD higher* = physician rated patient higher than patient themselves; *MD lower* = physician rated patient lower than patient themselves

^a^ – reference was male

* Bonferroni correction applied

### Change in agreement over time

Out of the 347 patients with data for baseline and 12-month agreement on function, 196 (56%) showed no change in agreement, 89 (26%) improved (went from disagreeing to agreeing) and 62 (18%) worsened. Compared to men, women had a higher probability of having a worse agreement on function over time (OR = 1.877, 95% CI 1.055–3.341, *p* = 0.032), being younger was close to being significantly associated with improvement in agreement on function over time (OR = 0.987, 95% CI 0.987–1.001, *p* = 0.066).

### Agreement on pain

#### Baseline agreement

Out of 647 patients with self-reported and physician-reported scores, 296 (45.7%) disagreed with their physician on the level of pain at baseline. Of those that disagreed, in 54.7% (162/296) of cases physician rated patients’ pain as worse than patients themselves, and in 45.3% (134/296) of cases physicians rated patients’ pain as lower than patients themselves.

#### Agreement and 12-month outcomes

Three adjusted mixed models were run to assess the association between the baseline agreement on pain, change in agreement over time, and their interaction with the main outcomes of interest (i.e., daily functioning, quality of life, and fatigue) at 12-months. Model 4 showed a significant main effect of time (*F*(1, 201) = 15.36, *p* < 0.001), and baseline agreement (*F*(2, 209) = 3.16, *p* = 0.044). Daily function significantly improved over time (β = 4.44, SE = 1.13, *p* < 0.001). Post-hoc analyses revealed no significant difference between different levels of baseline agreement and daily functioning.

Model 5 showed a main effect of time (*F*(1, 201) = 48.06, *p* < 0.001), baseline agreement (*F*(2, 200) = 4.20, *p* = 0.016), age (*F*(1, 203) = 3.92, *p* = 0.049, and a significant interaction of time and change (*F*(2, 201) = 3.21, *p* = 0.042) on 12-month scores of quality of life. Overall, quality of life significantly improved over time (β = 8.95, SE = 1.29, p < 0.001). Older age was associated with a slightly better quality of life (β = 0.1, SE = 0.05, p = 0.049). Patients who agreed with their physician at baseline reported significantly better quality of life than those patients where the physician rated their level of pain as lower than the patient themselves (β = -11.08, SE = 3.85, *p* = 0.013).

**Fig. 2:**
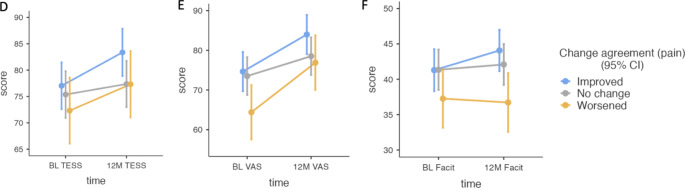
Agreement on pain: Time x change in agreement *BL = Baseline; 12 M = 12-months; TESS = Toronto Extremity Salvage Score; FACIT = Functional Assessment of Chronic Illness Therapy; VAS = Visual Analog Scale*.

Model 6 revealed a main effect of change in agreement over time (*F*(2, 207) = 4.41, *p* = 0.013). Patients with higher tumor grade reported significantly more fatigue (β = -1.51, SE = 0.62, *p* = 0.016). Also, patients with worsened agreement on pain over time reported significantly more fatigue (β = 4.73, SE = 1.60, *p* = 0.011, see Fig. 2). Complete model parameters can be found in Table 3 and the Supplementary materials. Figure 2 shows the change in agreement on pain and the main outcomes.

**Table 3:** Mixed models for agreement on pain

Fixed effects	Model 4: Daily function (TESS)			Model 5: Quality of life (VAS)			Model 6: Fatigue (FACIT)		
	Estimate (SE)	*t*	*p*	Estimate (SE)	*t*	*p*	Estimate (SE)	*t*	*p*
Intercept	77.12 (1.32)	74.53, 79.71	**< 0.001**	75.33 (1.44)	72.51, 78.14	**< 0.001**	40.46 (0.88)	38.74, 42.19	**< 0.001**
Time (BL)	4.44 (1.13)	2.22, 6.66	< 0.001*	8.95 (1.29)	6.42, 11.48	< 0.001*	0.99 (0.68)	-0.34, 2.32	0.146*
Gender^a^	0.85 (1.80)	-2.68, 4.37	0.638	-2.66 (1.97)	-6.53, 1.21	0.179	0.53 (1.22)	-1.86, 2.91	0.666
Age	0.05 (0.05)	-0.05, 0.14	0.318	0.10 (0.05)	0.00, 0.21	**0.049**	0.01 (0.03)	-0.06, 0.07	0.84
Education	0.55 (1.15)	-1.70, 2.81	0.63	0.59 (1.26)	-1.89, 3.07	0.641	0.56 (0.78)	-0.96, 2.09	0.468
Tumor grade	-0.26 (0.93)	-2.08, 1.55	0.776	-1.26 (1.02)	-3.25, 0.73	0.217	-1.51 (0.62)	-2.74, -0.29	**0.016**
**Baseline agreement**									
MD lower (ref) – MD higher	-0.54 (2.94)	-0.19	1.00*	4.13 (3.22)	1.28	0.604*	2.91 (1.98)	1.47	0.43*
Agree (ref) – MD higher	-8.81 (3.83)	-2.30	0.068*	-6.95 (4.13)	-1.68	0.281*	-1.99 (2.53)	-0.784	1.0*
Agree (ref) – MD lower	-8.26 (3.57)	-2.32	0.064*	-11.08 (3.85)	-2.88	0.013*	-4.89 (2.37)	-2.06	0.121*
**Change in agreement**									
No change (ref) – Improved	3.83 (3.54)	1.082	0.841*	3.30 (3.82)	0.87	1.0*	0.96 (2.35)	0.41	1.0*
Worsened (ref) - Improved	5.38 (4.30)	1.25	0.636*	8.67 (4.67)	1.86	0.193*	5.69 (2.87)	1.99	0.145*
Worsened (ref) – No change	1.55 (2.37)	0.66	1.0*	5.37 (2.60)	2.06	0.121*	4.73 (1.60)	2.95	0.011*
**Random effects**				**Random effects**			**Random effects**		
ICC	0.466			ICC	0.440		ICC	0.57	
Residual variance	115			Residual variance	148		Residual variance	39.2	

*NOTE*: *ref* = reference category, meaning that the estimate refers to that in comparison to the other; *MD higher* = physician rated patient higher than patient themselves; *MD lower* = physician rated patient lower than patient themselves

^a^ – reference was male

* Bonferroni correction applied

### Change in agreement over time

Out of the 330 patients with data for baseline and 12-month agreement on pain, 167 (51%) showed no change in agreement, 93 (28%) improved and 70 (21%) worsened. Compared to men, women had a higher probability of having a worse agreement on pain over time, although the association was marginal (OR = 1.729, 95% CI 0.985–3.034, *p* = 0.056), and lower education level was close to being significantly associated with the worsening in agreement on pain over time (OR = 0.658, 95% CI 0.459–1.02, *p* = 0.064).

## Discussion

Our study looked at the patient-physician agreement on the level of function and pain in a prospective sample of sarcoma patients. Overall, our findings demonstrate that patient-physician agreement matters; patients who agreed at baseline and those with improved agreement over time reported better outcomes – functioning, quality of life, and fatigue at 12-months.

We first considered agreement at baseline and found that 43.4% and 45.7% of patient-physician dyads disagreed on the level of function and pain, respectively. Interestingly, in both cases the disagreement showed a similar pattern; in 57.3% and 54.7% of cases physicians rated patients as worse on function and pain, while just under a half of the discordant dyads showed the inverse (see Supplementary materials). Previous studies that looked at patient-physician agreement on function based on performance status (PS) found similar rates of discordance, from 32,9% in advanced lung and colorectal cancer patients [16] to 50% in lung cancer patients [12]. Studies regarding patient-physician agreement on pain are limited, only one study about abdominal pain severity and the need for opioid analgesia revealed that physicians rated pain lower than patients, but had an overall good agreement (78.9%) on the need for opioids [38]. Other studies on patient-physician agreement concerned various topics, such as patients’ well-being, content discussed during consultations and shared-decision making [39–41]. Similarly, disagreements were common and underlined the need for better communication. Few studies identified possible factors for these discrepancies and found that consulting the physician for the first time, seeing different physicians during follow-up, [42] or not speaking the same language, [43] all played an important role.

A clear and significant pattern was revealed when considering the agreement on function. Notably, all outcomes; daily functioning, quality of life, and fatigue improved over time as is expected for most patients 12-moths after initial surgery. More interesting is the fact that baseline patient-physician agreement on function significantly predicted all outcomes at baseline and at 12-months. Moreover, change in agreement showed a significant pattern; those with improved agreement reported better outcomes. A possible explanation for this might be that understanding the patient better, i.e. agreement at baseline or an improved agreement over time, results in a more tailored healthcare based on the patients’ needs. For example, if a patient needs physiotherapy after surgery, but there is a disagreement on the function, the patient might not be referred accordingly, which may subsequently lead to lower daily functioning and quality of life.

Significant interaction of time and change in agreement was observed only in daily functioning and quality of life, but not fatigue. This is not completely unexpected, as the condition and physical functioning usually improves during the 12 months after surgery, patients get more active and seek the ‘normal’ life, resulting in more or less stable levels of fatigue. Additionally, the level of fatigue is not only influenced by physical, but also psychological factors [44, 45].

When considering agreement on pain, the pattern is not as clear. A possible reason might be that levels of pain were not taken into account while performing our analysis. Therefore, patients with various levels of pain were divided over the different agreement groups which may have resulted in a less clear pattern. Though the same applies to the analyses on function, pain might be more influential on daily function and quality of life compared to function [46–48]. Interestingly, quality of life was significantly higher for patients where physicians rated them as having less pain than the patient themselves. Persistent fatigue was associated with higher tumor grade, likely reflecting a higher disease and treatment burden. Fatigue is also the only outcome that worsened over time in case of patient-physician disagreement at 12-months. Fatigue is common and one of the most debilitating long-term sequalae of cancer [49]. Though its etiology is likely to be multifactorial, [50] our findings suggest that patient-physician communication may also play an indirect role.

### Clinical implications

Taken together the findings suggest that patient-physician agreement, which may be a proxy of communication, play a role in the patient pathways. Effective communication is important for creating therapeutic alliance, facilitating exchange of information, and promoting shared decision-making, thus also contributing to patients’ health literacy [51]. Good communication has also been associated with better emotion regulation, better identification of patients’ needs, better treatment adherence, as well as recovery [52]. These findings carry important clinical and public health implications. As patients navigate increasingly complex care, physicians and healthcare providers share a responsibility to guide patients, which can in turn improve health literacy, particularly in case of sarcomas, a rare disease. Beyond being proactive in one’s care, healthy literacy also implies a mutual understanding of outcomes, and open discussion about one’s needs [53]. However, agreement on patient outcomes is still seldom achieved; previous work has shown that the majority of patients were not aware that their opinions differed from their oncologists regarding their clinical prognosis, [13] and when asked, 75% of the orthopedic surgeons believed that they communicated satisfactorily, but only 21% of the patients agreed [54]. Going forward, communication and shared decision-making may benefit from including shared outcomes’ assessment. Although lack of time remains a limitation, this could be overcome by involving a wider team of healthcare providers that could further understand the nature of discordant reports.

### Study limitations

Our study is the first to investigate the patient-physician agreement in sarcoma patients, and even more unique in investigating a change in patient-physician agreement over time. We employed a relatively large prospective sample, and used mixed modelling, allowing us to capture individual heterogeneity and account for the correlated structure in our data. However, the models assume that data is missing at random, and though we performed sensitivity analyses this remains a limitation. The data also come from a single sarcoma specialty centre and thus limit generalizability to other sites and countries particularly because the communication is likely to depend on cultural context, as well and the healthcare system. Further, though the sample is relatively large for a rare type of cancer, we did not have adequate statistical power for further stratification by age, or tumor type. Also, the data on physician factors was missing and hard to include due to confidentiality. Future work should consider physician-level factors, particularly noting the significant sex differences observed in agreement over time.

Another important limitation is measurement of the agreement. The ratings were taken from two different questionnaires and harmonized via triangulation. The range of scale is limited to three points, which might have contributed to loss of granular information. Future work should employ the same scales. Furthermore, it would be interesting to ask physicians to rate patients’ outcomes such as daily functioning, quality of life, and fatigue. This could provide further insight into where the agreement and communication could improve the most.

Lastly, it is well established that psychological factors and mental health play an important role in patient-reported outcomes and might influence patient-physician communication, too. Our study did not account for psychological distress and further work should aim to explore the levels of agreement and its relationships mental health functioning.

## Conclusions

This longitudinal study offers novel insights into the way patient-physician agreement can play a role in long-term patient-reported outcomes in sarcoma. Patient-physician disagreement on the level of function and pain is common. Although most patients generally improve in terms of daily functioning, quality of life and fatigue over time, patient-physician agreement from baseline contributed to better scores. Being female, older, less educated and having a higher tumor grade may potentially all contribute to poorer levels of agreement between the patient and physician, and subsequently compromise patients’ daily functioning, quality of life, and even fatigue.

## Data Availability

Data are not available.
